# Growth hormone receptor promotes breast cancer progression via the BRAF/MEK/ERK signaling pathway

**DOI:** 10.1002/2211-5463.12816

**Published:** 2020-05-06

**Authors:** Xiaojue Zhu, Yonghao Li, Guoxin Xu, ChangQing Fu

**Affiliations:** ^1^ Clinical Laboratory Zhangjiagang First People’s Hospital Suzhou University Suzhou China; ^2^ Zhangjiagang Fifth People's Hospital Suzhou China

**Keywords:** apoptosis, BRAF/MEK/ERK signaling pathway, breast cancer, cell cycle, GHR

## Abstract

Growth hormone receptor (GHR), a member of the class I cytokine receptor family, plays key roles in cancer progression. Recently, GHR has been reported to be associated with breast cancer development, but the molecular mechanism of GHR in this malignancy is not fully understood. To investigate this issue, we stably inhibited GHR in breast cancer cell lines, which were observed to reduce cell proliferation, tumor growth and induction of apoptosis, and arrest the cell‐cycle arrest at the G1–S phase transition. In addition, GHR silencing suppressed the protein levels of B‐Raf proto‐oncogene, serine/threonine kinase (BRAF), Mitogen‐activated protein kinase kinase (MEK) and Extracellular regulated protein kinases (ERK). These findings suggest that GHR may mediate breast cell progression and apoptosis through control of the cell cycle via the BRAF/MEK/ERK signaling pathway.

## Graphical abstract

Here, we stably inhibited growth hormone receptor (GHR) in breast cancer cell lines, which reduced cell proliferation, tumor growth and induction of apoptosis, and arrested the cell cycle at the G1–S phase transition. In addition, GHR silencing reduced the protein levels of B‐Raf proto‐oncogene, serine/threonine kinase (BRAF), Mitogen‐activated protein kinase kinase (MEK) and Extracellular regulated protein kinases (ERK). These findings suggest that GHR may mediate breast cell progression and apoptosis through control of the cell cycle via the BRAF/MEK/ERK signaling pathway.

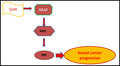

**Abbreviations**
CDK: cyclin‐dependent kinase
GH: growth hormone
GHR: growth hormone receptor
IGF‐I: insulin‐like growth factor I
JAK2: Janus Kinase 2
p‐: phosphorylated
PARP: poly (ADP‐ribose) polymerase
PI: propidium iodide
SEM: standard error of the mean

Breast cancer is the most common cancer in women and the second leading cause of death from cancer. Although early diagnosis and treatment prevent cancer progression and decrease its morbidity rates in recent decades, the survival rate is still low in less developed countries. Breast cancer is a heterogeneous disease that has a variable response to treatment, and differences in prognosis block its management 1. Targeted therapy is an effective treatment strategy. The well‐known markers for breast cancer include human epidermal growth factor receptor 2, mammalian target of rapamycin signaling pathway, vascular endothelial growth factor, epithelial growth factor receptor, poly (ADP‐ribose) polymerase (PARP) and cyclin‐dependent kinase 4/6 (CDK 4/6) 2. However, novel candidates for targeted therapy of breast cancer still need to be discovered.

Growth hormone, a peptide hormone produced in the anterior pituitary gland, induces cell division, regeneration and growth 3, 4. It first binds to preformed growth hormone (GH) receptor (GHR) dimers and then causes a conformational change, further activating the GHR, JAK2 (Janus Kinase 2) and STAT5 signaling pathway 5, 6, 7 and inducing the synthesis of insulin‐like growth factor I (IGF‐I) in liver 8, 9, 10. The effect of the GH/IGF‐1 system on cancer progression recently has been the focus of much interest. GH/IGF‐1 axis dysregulation enhances the synergistic effect of GH and IGF‐1 on the promotion of uncontrolled cell proliferation, cell movement and angiogenesis, as well as the increase of neoplasia risk 11. Adult height has been used as a biomarker of GH and IGF‐1 action, which is an independent risk for malignancy 9. Recently, it was also reported that GHR modulates osteosarcoma cell proliferation and metastasis through the phosphoinositide 3‐kinase/AKT signaling pathway 12. Previous studies have reported that individuals taller than 175 cm have a 20% higher risk for prostate cancer, a 22% higher risk for breast cancer and a 20–60% higher risk for colorectal cancer compared with control subjects shorter than 160 cm 13, 14.

The GHR, a member of the class I cytokine receptor family, exists as a constitutive dimer in the cell membrane 9. It is well‐known that GHR is involved in growth regulation. Other important biological functions include metabolism regulation and physiological processes control in hepatobiliary, cardiovascular, renal, gastrointestinal and reproductive systems 11. GHR is widely distributed in various types of normal and tumor cells with different expression levels, and plays an important role in cancer progression 15. In addition, it is also involved in breast cancer development and progression because its expression is reported to be increased in breast cancer compared with the adjacent normal tissue 16. A previous study has demonstrated that GHR deficiency results in reduced risk for death from cancer, suggesting that GHR may be used as a therapeutic target for cancer treatment 17. GHR silencing also inhibits GH‐induced chemoresistance in breast cancer cells with positive estrogen receptor 18. In addition, overexpression of GHR is found to enhance chemoresistance and metastasis of estrogen receptor–negative breast cancer 19. These reports suggest that GHR may be a potential therapeutic target for breast cancer and GH‐induced chemoresistance.

To investigate the molecular mechanism of GHR in breast cancer, this study evaluated the biological functions of GHR in breast cancer *in vitro* and *in vivo*, and explored the association between GHR and the B‐Raf proto‐oncogene, serine/threonine kinase (BRAF)/Mitogen‐activated protein kinase kinase (MEK)/Extracellular regulated protein kinases (ERK) signaling pathway. Our results showed that GHR reduction led to the inhibition of breast cancer cell lines proliferation and tumor growth, the induction of cell apoptosis and the cell‐cycle arrest in G1–S phase transition. All of these changes might be due to the inhibition of the BRAF/MEK/ERK signaling pathway caused by GHR silencing.

## Materials and methods

### Breast cancer tissues

A total of 12 breast cancer tissues and 12 adjacent normal tissues were collected from patients with breast cancer who underwent surgery at Zhangjiagang First People’s Hospital. Tissues were frozen in liquid nitrogen and stored at −80 °C. Our research was approved by the Ethics Committee of Zhangjiagang First People’s Hospital, and written informed consent was obtained from each patient. The study methodologies conformed to the standards set by the Declaration of Helsinki.

### Cell lines and culture

Nonmalignant (MCF10A, MMuMG) and malignant (MDA‐MB‐468, MCF‐7 and MDA‐MB‐231) breast cell lines were obtained from the American Type Culture Collection (ATCC, Manassas, VA, USA). Nonmalignant breast cell lines were cultured in mammary epithelial cell growth medium with supplements (Lonza, Walkersville, MD, USA), and malignant breast cell lines were cultured in RPMI 1640 medium (Thermo Scientific, Rockford, IL, USA) supplemented with 10% FBS (Gibco, Gaithersburg, MD, USA) at 37 °C in an atmosphere of 95% air and 5% CO_2_.

Stable cell lines of MDA‐MB‐231 were generated by integration of retroviral shRNA vectors specific for GHR or a control vector from OriGene (Rockville, MD, USA).

### Quantitative RT‐PCR

Total RNA was extracted by using TRIzol reagent (TaKaRa Bio, Kusatsu, Japan) and was reverse transcribed into cDNA by using a First‐Strand cDNA Synthesis kit (TaKaRa Bio). SYBR mixed with cDNA was used to perform quantitative RT‐PCR. The relative expression level of GHR in each type of cell was analyzed by using the$2^{-\Delta \Delta C_{t}}$method. Primers were listed as: GHR forward, 5′‐GCAACCAGAUCCACCCAUUTT‐3′; reverse, 5′‐AAUGGGUGGAUCUGGUUGCTT‐3′.

### Cell transfection and siRNA

Cells were transected with siRNA plasmids of GHR by using Lipofectamine 2000 transfection reagent (Life Technologies, Grand Island, NY, USA). Cells were cultured in six‐well plates overnight and transfected with 5 μg plasmids mixed with Lipofectamine solution. Cells were then collected and analyzed by western blot to confirm silencing of GHR expression. The two siRNAs that targeted GHR were 5′‐GCAACCAGAUCCACCCAUUTT‐3′ and 5′‐GCACCACGCAAUGCAGAUATT‐3′.

### Western blot

Total proteins from cells were extracted using lysis buffer and were separated on 10% SDS/PAGE gels. Proteins were then transferred from gels to polyvinylidene difluoride membranes, which were further blocked with 5% BSA and incubated with primary antibodies overnight at 4 °C. Subsequently, membranes were incubated with diluted secondary goat polyclonal anti‐rabbit IgG (1 : 2000; Abcam, Cambridge, MA, USA). Protein levels were evaluated using an enhanced chemiluminescence system. Antibodies were purchased from Proteintech (Rosemont, IL, USA; GHR), Sigma (St. Louis, MO, USA; β‐actin) and Cell Signaling Technology (Danvers, MA, USA; p‐BRAF, BRAF, p‐MEK, MEK, p‐ERK, ERK, p‐JAK2, JAK2, p‐STAT5, STAT5, IR DyeR 800 goat anti‐Mouse, IR DyeR 800 goat anti‐Rabbit).

### Colony formation assay

Colony formation assay was performed to detect clonogenic ability of a single cell. After transfection with siGHR, cells were stained with crystal violet; then colonies of cells were counted.

### Apoptosis assay

The number of apoptotic cells was assessed by using Annexin V–FITC Apoptosis Detection Kit I (BD Biosciences, San Jose, CA, USA). Cells were incubated with propidium iodide (PI) and Annexin V–FITC staining for 20 min in the dark; then cell apoptosis was analyzed on a flow cytometer (BD Biosciences, Franklin Lakes, NJ, USA). The extent of apoptosis was further quantified as a percentage of Annexin V–FITC‐positive cells.

### Cell‐cycle analysis

Cells were collected and stained with PI and ribonuclease according to the manufacturer’s instructions at room temperature overnight. Flow cytometer was used to determine DNA PI‐associated fluorescence in cells.

### Xenograft mouse model analysis

BALB/c nude mice were subcutaneously injected with 2 × 10^6^ MDA‐MB‐231 cells or shGHR stable cells in the right flank for 4 weeks. Tumor volume was measured twice a week by using a digital caliper. At last, mice were sacrificed, and tumor weight was detected.

### Ethics statement

All experimental protocols and methods were approved by Zhangjiagang Fifth People’s Hospital (No. 20190705). We also confirmed that all methods were performed in accordance with the relevant guidelines and regulations. Mice were bred in the Animal Core Facility by following procedures approved by Zhangjiagang Fifth People’s Hospital of Institutional Animal Care and Use Committee.

### Statistical analysis

Each experiment was conducted in three replicates. The data were expressed as the mean ± standard error of the mean (SEM). Statistics analysis was performed with spss software (Version X; IBM, Armonk, NY, USA), and Student’s *t*‐test was used to evaluate individual differences between means. A *P* value <0.05 was considered significant.

## Results

### GHR was highly expressed in breast cancer

**Fig. 1:**
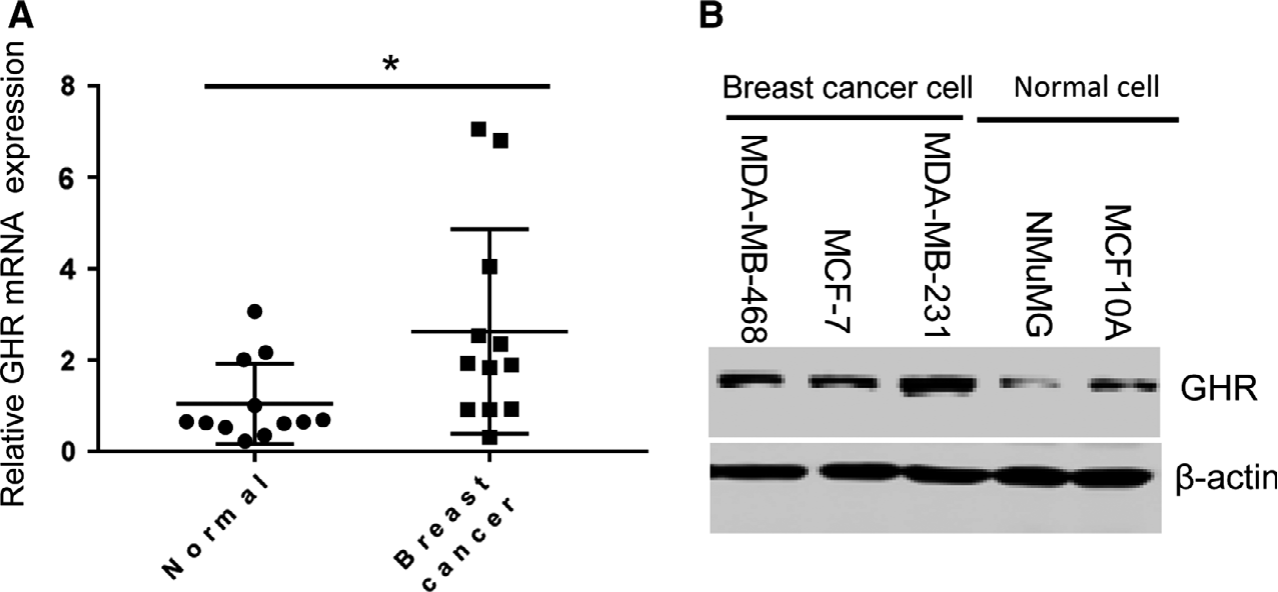
The expression levels of GHR in breast cancer tissues and cell lines. (A) GHR was highly expressed in breast cancer tissues compared with adjacent peritumoral tissues. (B) GHR level was significantly increased in breast cancer cell lines (MDA‐MB‐468, MCF‐7 and MDA‐MB‐231) compared with nonmalignant breast cell lines (NMuMG and MCF10A). *P < 0.05. Data represent means ± SEM, as determined by Student’s *t*‐test.

To determine GHR expression, this study analyzed 12 breast cancer tissues and 12 adjacent normal tissues. Overexpression of GHR’s mRNA was observed in clinical tumors compared with normal tissues. In addition, we also detected GHR protein levels *in vitro* (Fig. 1A). Three breast cancer cell lines, including MDA‐MB‐468, MCF‐7 and MDA‐MB‐231, were further used. Western blotting assay showed that GHR protein levels were increased in breast cancer cells relative to normal cells (NMuMG and MCF10A) (Fig. 1B).

### Silencing GHR significantly inhibited breast cancer cell proliferation

**Fig. 2:**
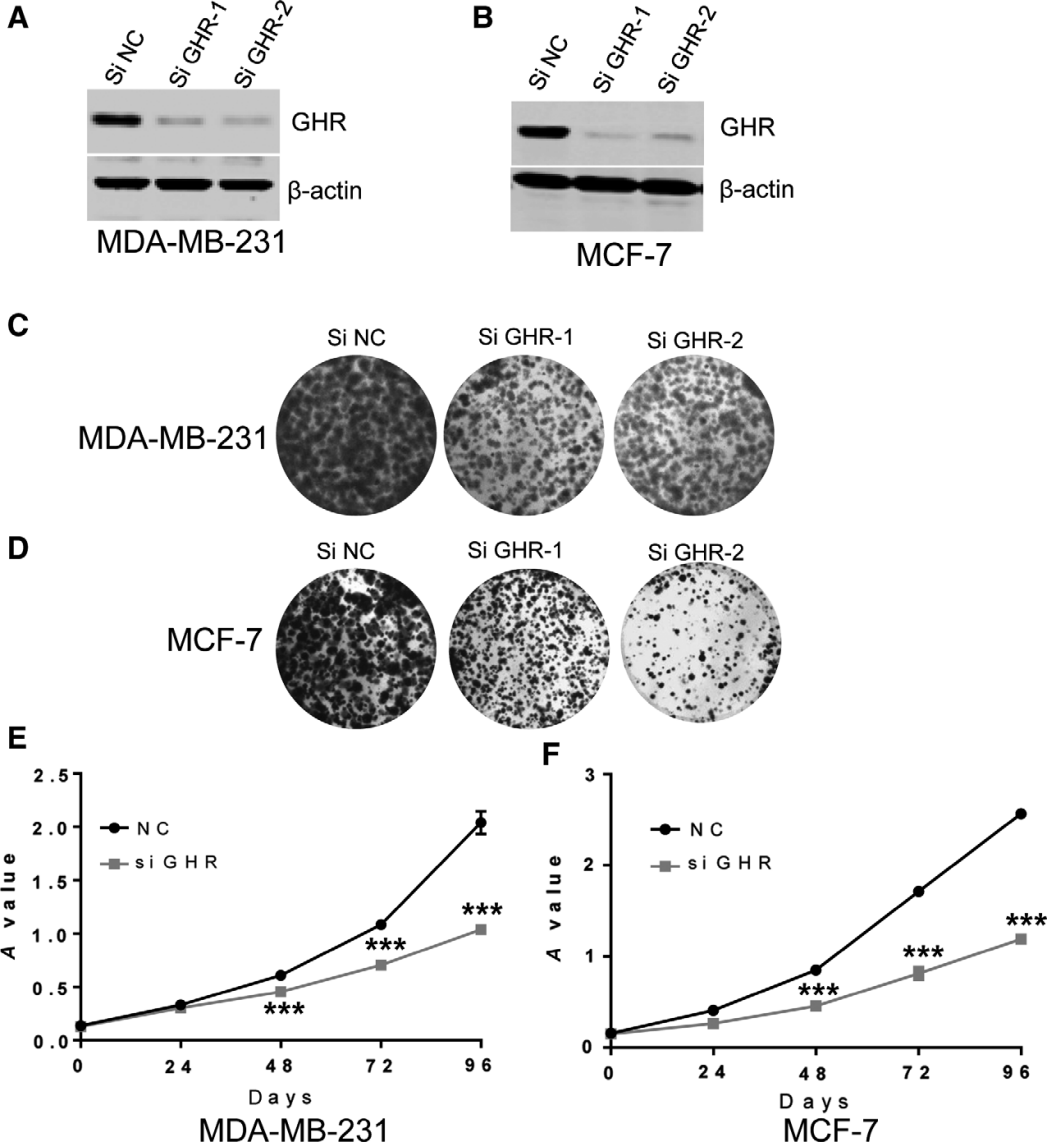
The influence of GHR on the growth of breast cancer cells. The expression of GHR knockdown was detected by western blotting. The results showed that GHR expression was significantly decreased in MDA‐MB‐231 cells (A) and MCF‐7 cells (B) with siGHR transfection. (C, D) Control and GHR knockdown MDA‐MB‐231(C) and MCF‐7 cells (D) were analyzed by colony formation. (E, F) Control and GHR knockdown MDA‐MB‐231 (E) and MCF‐7 cells (F) were examined for cell proliferation ability by 3‐(4,5‐dimethylthiazol‐2‐yl)‐2,5‐diphenyl‐tetrazolium bromide assay. Data represent means ± SEM; ***P < 0.001, as determined by Student’s *t*‐test. All experiments were repeated three times (*n* = 3).

To further investigate the roles of GHR in breast cancer progression, this study blocked GHR expression by using siRNA transfection. Western blotting assay showed that GHR protein level was successfully suppressed in MDA‐MB‐231 and MCF‐7 cells (Fig. 2A,B). Cell proliferation of the stably transfected cells was determined by clonogenic assay (Fig. 2C,D). In addition, GHR inhibition induced significant decrease of breast cancer cell growth by 3‐(4,5‐dimethylthiazol‐2‐yl)‐2,5‐diphenyl‐tetrazolium bromide assay (Fig. 2E,F). These findings suggested that GHR silencing inhibited cell proliferation.

### GHR knockdown induced MDA‐MB‐231 and MCF‐7 cell apoptosis and cell‐cycle arrest

**Fig. 3:**
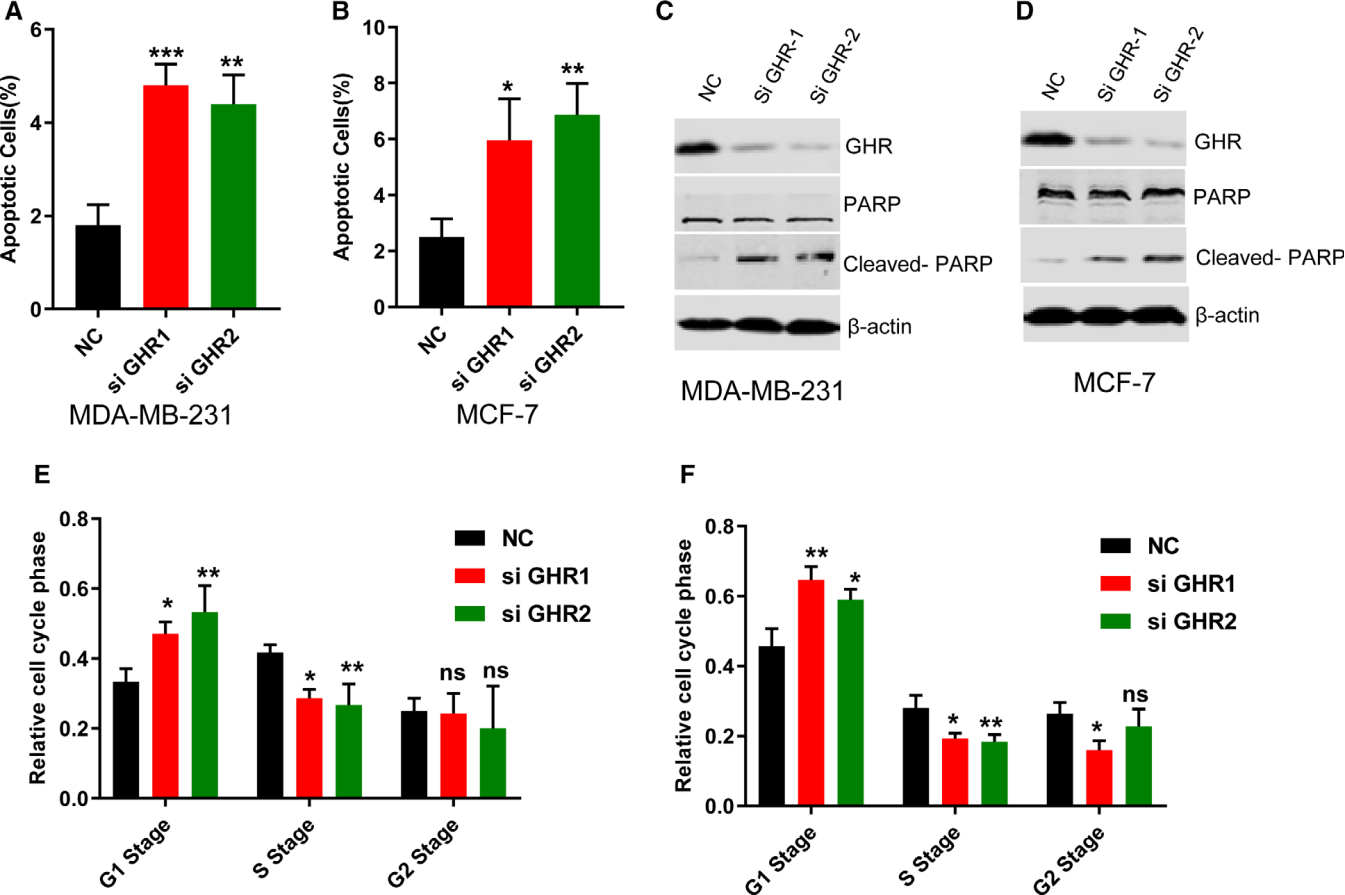
The impact of GHR on the apoptosis of breast cancer cell and cell cycle. The apoptosis was analyzed by PI and Annexin V staining in control and GHR knockdown MDA‐MB‐231 (A) and MCF‐7 (B) cells. Data represent mean ± SEM (*n* = 3); two‐tailed Student’s *t*‐test was used for statistical analysis, **P < 0.01; ***P < 0.001. The reduction of GHR significantly induced the apoptosis of MDA‐MB‐231 and MCF‐7 cells. (C) GHR inhibition stimulated the protein levels of cleaved PARP in MDA‐MB‐231 cells (C) and MCF‐7 cells (D). GHR inhibition caused the arrest of cell cycle in G1–S transition (E, F). *P < 0.05. **P < 0.01. ns, not significant.

To investigate the role of GHR in breast cancer cell apoptosis, we further performed apoptosis assay by PI and Annexin V staining. The results showed that silencing of GHR significantly increased cell apoptosis in both MDA‐MB‐231 and MCF‐7 cells (Fig. 3A,B), which might be the reason for cell proliferation inhibition caused by GHR silencing. This study then found that GHR inhibition stimulated the protein levels of cleaved PARP (Fig. 3C,D), one of the most used diagnostic tools for the detection of apoptosis. PARP, an abundant DNA‐binding enzyme that detects and signals DNA strand breaks, is the main substrate cleaved by caspase‐3 and caspase‐7 20. Thus, cleaved PARP is a valuable marker of apoptosis.

In addition, the progression through cell cycle was evaluated by cytofluorimetry in both MDA‐MB‐231 and MCF‐7 cells with siGHR. GHR blockade induced the accumulation of MDA‐MB‐231 and MCF‐7 cells in the G1 phase of the cell cycle, whereas it caused a decrease of cell growth in the S phase, suggesting that GHR deficiency prevented G1‐to‐S phase progression (Fig. 3E,F). In G2 phase, GHR suppression did not affect MDA‐MB‐231 cell growth, and only siGHR1 inhibited MCF‐7 cell growth (Fig. 3E,F). These findings suggested that the inhibition of cell growth and the stimulation of cell apoptosis caused by GHR blockade might be due to modifications in cell‐cycle progression in breast cancer cell lines.

### GHR was involved in the BRAF/MEK/ERK and JAK/STAT signaling pathways

**Fig. 4:**
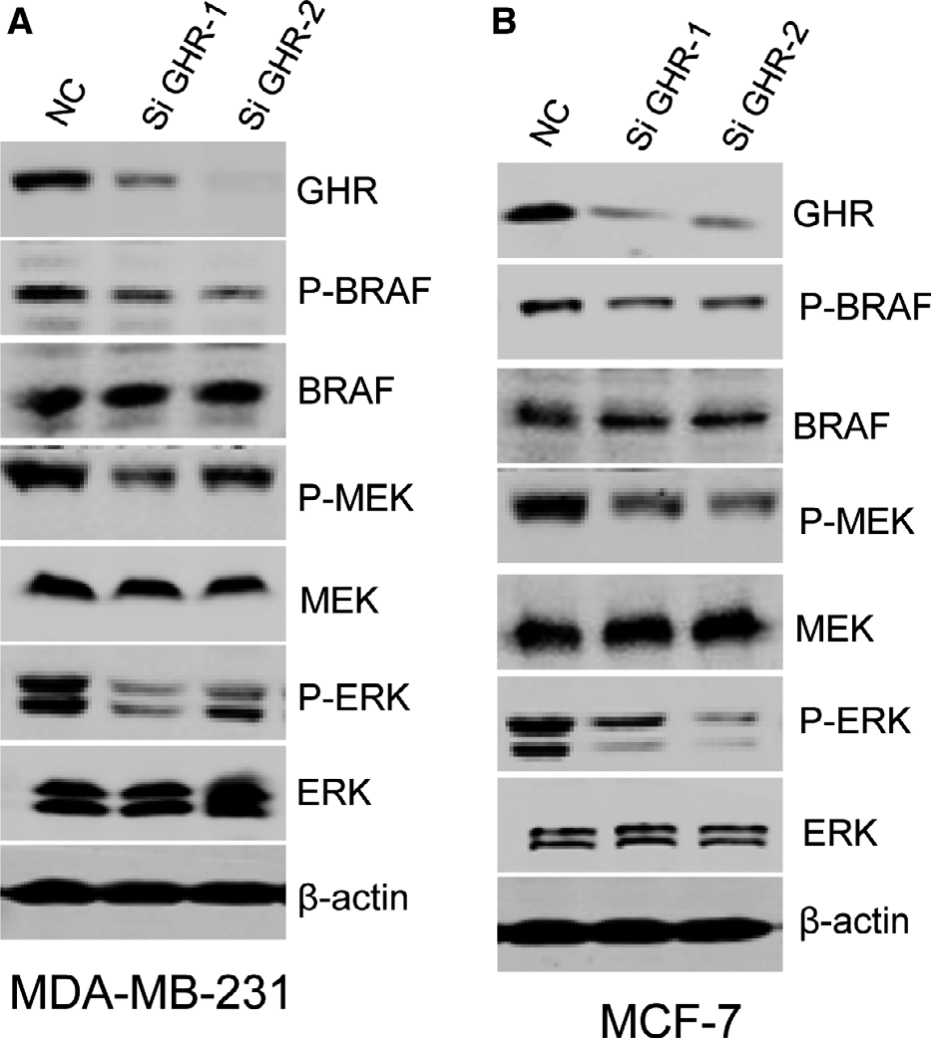
GHR was involved in the BRAF/MEK/ERK signaling pathway. (A) Inhibition of GHR significantly reduced the protein levels of p‐BRAF, p‐MEK and p‐ERK in both MDA‐MB‐231 cell lines. (B) Similar results were observed in the MCF‐7 cell line.

To verify whether GHR is associated with breast cancer progression by modulating the BRAF/MEK/ERK signaling pathway, we evaluated the roles of GHR silencing in the expression of p‐BRAF, p‐MEK and p‐ERK. Inhibition of GHR significantly reduced the protein levels of p‐BRAF, p‐MEK and p‐ERK in both MDA‐MB‐231 cell lines. Similar results were observed in the MCF‐7 cell line (Fig. 4A,B). GHR has long been known to use the JAK/Signal transducers and activators of transcription (STAT) signaling pathway to signal, and we also defined that GHR activated JAK2/STAT5 signaling (Fig. S1A,B).

### GHR silencing led to tumor weight and volume decrease in the xenograft mouse model

**Fig. 5:**
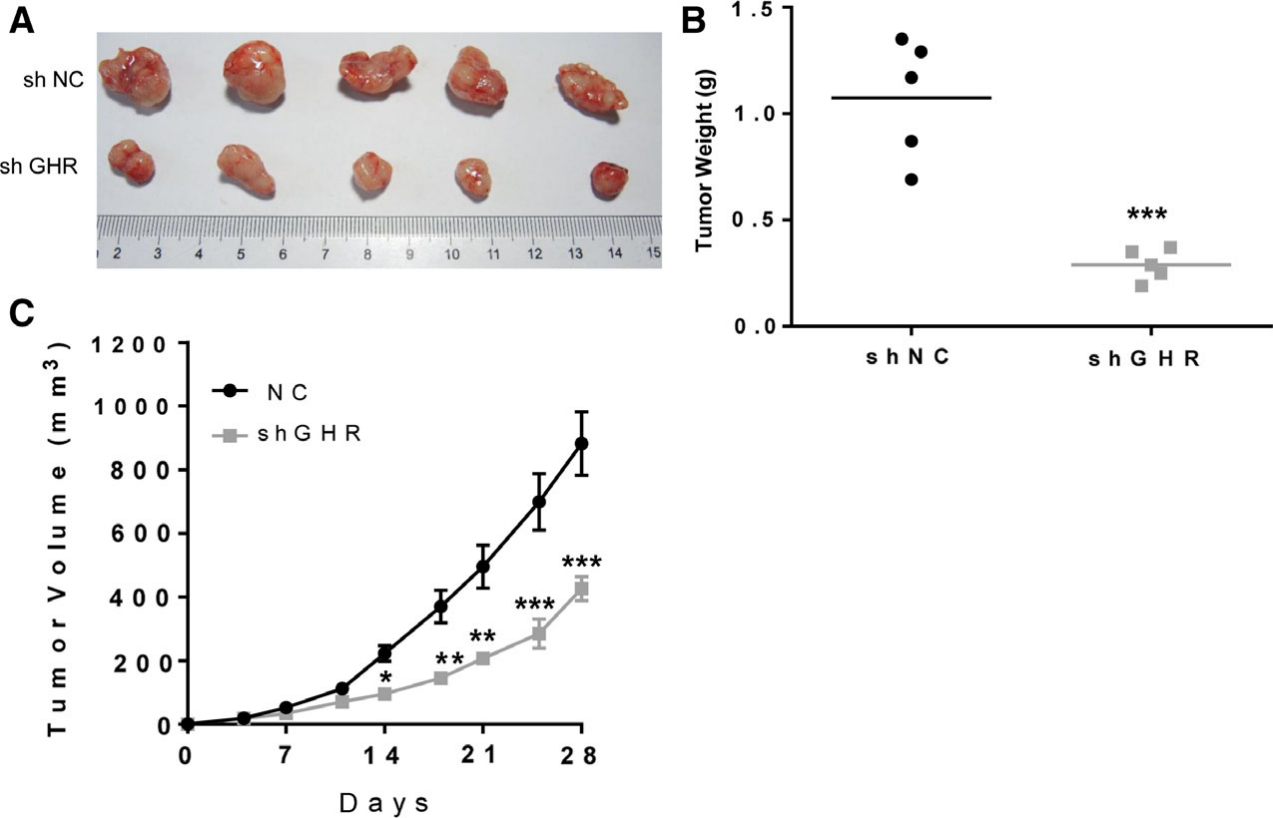
GHR reduction inhibited tumor growth in the xenograft MDA‐MB‐231 tumors. (A) Tumor size was smaller in mice established by MDA‐MB‐231 cells with shGHR transfection than that established by MDA‐MB‐231 cells. (B) GHR reduction inhibited tumor weight. (C) GHR reduction inhibited tumor volume. *P < 0.05; **P < 0.01; ***P < 0.001. Data represent means ± SEM, as determined by Student’s *t*‐test (*n* = 5).

MDA‐MB‐231 breast cancer cells with or without GHR silencing were subcutaneously injected into the right flank region of female mice to establish xenograft mouse models. GHR inhibition significantly reduced the tumor weight after 1 month compared with the vehicle control group (Fig. 5A,B). The tumor volume was also detected twice every week, and GHR silencing markedly decreased the tumor volume after 2 weeks (Fig. 5C). These results indicated that GHR promotes the proliferation of breast cancer cells *in vivo*.

## Discussion

GHR, a member of the class I cytokine receptor family, has been reported to be associated with breast cancer development and progression 16, 18, 19. Here, we attempted to find one mechanism underlying the influence of GHR on breast cancer progression. Our data showed that GHR deficiency significantly inhibited breast cancer cell lines and tumor growth, and induced cell apoptosis increase. Silencing GHR inhibited the activation of the BRAF/MEK/ERK signaling pathway, which might be one of the mechanisms underlying breast cancer progression inhibition caused by GH suppression.

In detail, GHR was highly expressed in tumor tissues from patients with breast cancer and breast cancer lines compared with normal control subjects, which is consistent with a previous study 16. The deficient GHR obviously inhibited cell proliferation in MDA‐MB‐231 and MCF‐7 cell lines, as well as tumor growth in xenograft mouse models established by MDA‐MB‐231 cells. In addition, we further demonstrated that deficient GHR in MDA‐MB‐231 and MCF‐7 cell lines caused apoptosis increase. Breast cancer cells were accumulated in the G1 phase of the cell cycle and then suddenly decreased in S phase caused by GHR inhibition, indicating GHR deficiency prevented G1‐to‐S phase progression. GHR inhibition stimulated the protein levels of cleaved PARP, an abundant DNA‐binding enzyme that detects and signals DNA strand breaks. These findings revealed that GHR deficiency inhibited cell proliferation by several mechanisms, including apoptosis induction, cell‐cycle blockade and DNA replication suppression. Similar results were obtained by Kaulsay *et al*. 21 and Pawlowski *et al*. 22.

JAK2 is regarded as the classical GHR signaling kinase 23. GHR has long been known to use the JAK/STAT signaling pathway to signal; however, more and more evidence has demonstrated that GHR is able to use additional pathways independent of JAK2, including the Src/ERK pathway 11, 24, 25. Barclay *et al*. 26 targeted knockin mutations to the box 1 sequence of GHR in mice, which inhibited JAK2 activation by GH *in vivo*, and found that the activation of hepatic ERK via Src did not decrease. Src family kinase is reported to associate constitutively with GHR, and acts in parallel to JAK2/STAT5, which activates RAS and then ERK1/2 5, 6, 25. The GHR/JAK2/STAT pathway has the most pronounced role in the somatotropic axis signal transduction. A previous study implicates the GH/GHR axis in inducing chemoresistance in human melanoma by JAK2/STAT5 activation 5. We present *in vitro* and *in vivo* evidence that GHR strongly drives the JAK2/STAT5 pathway in breast cancer progression in line with the report that the GHR/JAK2/STAT pathway plays a key role in the regulation of metabolic processes in an organism 6.

RAS and ERK are both involved in the mitogen‐activated protein kinases (MAPK) signaling cascade, whose components also include RAF and MEK 27. Pawlowski *et al*. 22 have reported that GHR inhibition reduced the expression of phosphorylated (p)‐ERK1/2, which is consistent with our results. To further investigate the role of GHR in the MAPK pathway, this study also detected the association between GHR reduction and the expression of RAF, as well as MEK. The MAPK signaling cascade is first triggered by RAS G protein activation, activating RAF and further causing mitogen‐activated protein kinase (MAPK) phosphorylation 28. This signaling pathway regulates cell‐cycle progression and apoptosis in diverse types of cells, and induces events related to both cell proliferation and cell‐cycle arrest 29. That significant reduction of GHR followed by inhibition of MAPK signaling might result in the arrest of cell cycle in G1–S transition and increased apoptosis. Cell cycle is mediated by a class of nuclear enzymes named CDKs, including CDK4 and CDK2, which regulate progression through G1 phase 30. MAPK signaling can regulate cell‐cycle progression through p21^Cip1^, an inhibitor of CDK2, which is poorly expressed in quiescent cells, but rapidly induced in early G1 phase through growth factor stimulation 30. It is reported that in BRAF‐transfected cells, p21^Cip1^ is significantly induced, leading to the inhibition of cell proliferation 29. ERK activation is reported to speed up the cell cycle in G1–S transition 31. In this study, GHR reduction inhibited the protein levels of BRAF, MEK and ERK, which might further induce p21^Cip1^ expression. However, this prediction needs to be confirmed in the future.

## Conclusion

On the basis of the results presented earlier, we conclude that GHR mediates breast cancer cell progression and apoptosis through controlling cell cycle in G1–S phase transition as a regulator of the BRAF/MEK/ERK signaling pathway. These findings give new insight to the roles of GHR in breast cancer.

## Conflict of interest

The authors declare no conflict of interest.

## Author contributions

XZ, GX, YL and CF participated in the design of the study, and performed the measurements and the statistical analysis. GX and YL helped in data collection and the interpretation of data. YL and CF wrote the manuscript. All authors read and approved the manuscript.

## Supporting information


**Fig. S1.** (A and B) The protein expression of p‐JAK2, JAK2, p‐STAT5 and STAT5 in MDA‐MB‐231 and MCF‐7 cells before and after RNA interference (RNAi) depletion of GHR was detected by western blotting.
