# Supplementary material for: Growth hormone receptor promotes breast cancer progression via the BRAF/MEK/ERK signaling pathway
Source: FEBS Open Bio. 2020 May 6;10(6):1013–20. doi: 10.1002/2211-5463.12816 (PMC7262926; doi:10.1002/2211-5463.12816)

Figure S1

A-B. The protein expression of P-JAK2, JAK2, P-STAT5 and STAT5 in MDA-MB-231 and MCF-7 cells before and after RNAi depletion of GHR was detected by Western blotting.

Supporting Figure 1

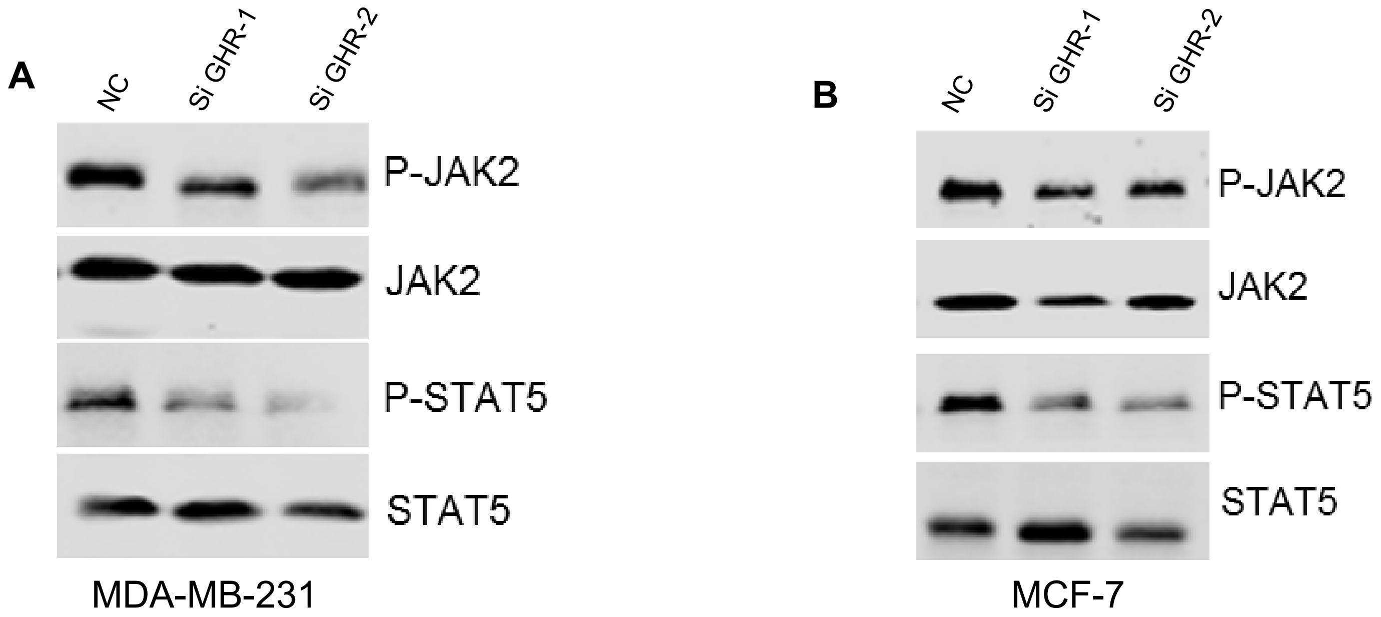

Supplement: Supplementary file 1 — Fig. S1. (A and B) The protein expression of p‐JAK2, JAK2, p‐STAT5 and STAT5 in MDA‐MB‐231 and MCF‐7 cells before and after RNA interference (RNAi) depletion of GHR was detected by western blotting. [file FEB4-10-1013-s001.pdf]
